# Supplementary material for: Intraday reliability, sensitivity, and minimum detectable change of national physical fitness measurement for preschool children in China
Source: PLoS One. 2020 Nov 20;15(11):e0242369. doi: 10.1371/journal.pone.0242369 (PMC7678998; doi:10.1371/journal.pone.0242369)
Supplement: Attachment — Submitted filename: Commends for the manuscript.docx [file pone.0242369.s002.docx]

Commends for the manuscript ”The intraday reliability, sensitivity and minimum detectable change of National Physical Fitness Measurement for Preschool Children in Chin”

Thank you for inviting me to review the manuscript on “The intraday reliability, sensitivity and minimum detectable change of National Physical Fitness Measurement for Preschool Children in China”. This research investigated the reliability, sensitivity and minimum detectable change values of NPFM in preschool children aged between 3.5 to 6 years. Overall the manuscript is well-written. I have a few minor comments that I would like the authors to consider.

1. In Abstract, Please provide more detail on the reliability and sensitivity analysis method.

2. Line 32, the keywords of “muscle strength; balance test” should be changed, ie. test-retest reliability...

3. In the Procedures, please clarify no previous familiarization was given for any test, although it was mentioned in line 342.

how much studies were identified in the review and how many provided data. Are there any differences in design or populations of those who provide data versus those who do not?

4. I feel confused there were two age groups (3.5-year, 4-year and 4.5-year vs. 5-year, 5.5-year and 6-year) in analyzing the intraday reliability, SEM, SWC, MDC95 and classification of sensitivity, on page 12 lines 208-237, why showed the results in table 3 & table 4 ?

5. I’m also confused the sentence, “To further improve the test-retest reliability of NPFM in preschoolers of different age groups or genders, researchers and practitioners should provide sufficient warm-up and practice opportunity to minimize learning effects”, What is meant by it. Which results could be deduced such the conclusion or advice in this manuscript?
