# Supplementary material for: Intraday reliability, sensitivity, and minimum detectable change of national physical fitness measurement for preschool children in China
Source: PLoS One. 2020 Nov 20;15(11):e0242369. doi: 10.1371/journal.pone.0242369 (PMC7678998; doi:10.1371/journal.pone.0242369)
Supplement: Attachment — Submitted filename: Commends for the manuscript _10-09-20.docx [file pone.0242369.s003.docx]

Commends for the manuscript ”The intraday reliability, sensitivity and minimum detectable change of National Physical Fitness Measurement for Preschool Children in Chin”

I agree with Hraski and the author's suggestion in line 405-406, which recommended using five trials for each testing item for preschool children to remove the potential learning effect from the first few attempts. However, I think the author's statement is difficult to understand and accept in abstract(line 37 to 40), which is “Hence, NPFM guidelines should be revised by adding adequate familiarization sessions and standardized warm-up protocols as well as increasing the number of testing trials. SWC and MDC95 values of NPFM tests should be considered to realize true performance changes”. The main purpose of this manuscript was to investigate the reliability, sensitivity and minimum detectable change values of NPFM in preschool children, there was few result supported the author's point. So, it should be deleted in the abstract.

In conclusion (line 437 to 444)，these sentences also should be deleted which were similar to the discussion section.
