# Supplementary material for: Intraday reliability, sensitivity, and minimum detectable change of national physical fitness measurement for preschool children in China
Source: PLoS One. 2020 Nov 20;15(11):e0242369. doi: 10.1371/journal.pone.0242369 (PMC7678998; doi:10.1371/journal.pone.0242369)
Supplement: Attachment — Submitted filename: Response to Reviewer 2.docx [file pone.0242369.s004.docx]

We thank the reviewers and the academic editor for their time and valuable suggestions. In this revised version we have answered all the questions raised by the reviewers and the editor. We hope that this revised manuscript meets the standard for publication in PLOS ONE. Below please find our point-to-point responses to reviewers.

Reviewer: 1

I agree with Hraski and the author's suggestion in line 405-406, which recommended using five trials for each testing item for preschool children to remove the potential learning effect from the first few attempts. However, I think the author's statement is difficult to understand and accept in abstract(line 37 to 40), which is “Hence, NPFM guidelines should be revised by adding adequate familiarization sessions and standardized warm-up protocols as well as increasing the number of testing trials. SWC and MDC95 values of NPFM tests should be considered to realize true performance changes”. The main purpose of this manuscript was to investigate the reliability, sensitivity and minimum detectable change values of NPFM in preschool children, there was few result supported the author's point. So, it should be deleted in the abstract.
In conclusion (line 437 to 444)，these sentences also should be deleted which were similar to the discussion section.

Response from authors: Deleted the line 37 to 40 of abstract and also line 437 to 444 of conclusion accordingly

Academic Editor:

In methods part of abstract, please also mention, when the study was conducted.

Response from authors: Added the time of test back to abstract
Also, please state the Six items of NPFM in the methods.

Response from authors: Added

In main text, please state what is the meaning of (ICC 3, 1) as the readers may not be familiar with it.

Response from authors: full form of ICC_3,1_ added as line 168 “intraclass correlation coefficient with two-way mixed-effects model and single measurement (ICC_3,1_)”

The heading under results in the result section (line 193-195) and another one in line 220-22 are too long. Please make it short and clear.

Response from authors: the heading is trimmed as requested
In discussion, please do not repeat the statistical results.

Response from authors: most statistical results originally included in the brackets were deleted per request. Only those essential in the main text for discussion purpose is retained.

The conclusion presented in the abstract and in the main texts should be aligned.

Response from authors: per the request of reviewer, we have deleted the last sentence to avoid redundant/duplicated sentences.
